# Gastric Cancer Risk and Pathogenesis in *BRCA1* and *BRCA2* Carriers

**DOI:** 10.3390/cancers14235953

**Published:** 2022-12-01

**Authors:** Kole H. Buckley, Blake A. Niccum, Kara N. Maxwell, Bryson W. Katona

**Affiliations:** 1 Division of Gastroenterology and Hepatology, University of Pennsylvania Perelman School of Medicine, 3400 Civic Center Blvd., Philadelphia, PA 19104, USA; 2 Division of Hematology and Oncology, University of Pennsylvania Perelman School of Medicine, Philadelphia, PA 19104, USA

**Keywords:** breast cancer susceptibility gene, DNA damage, hereditary breast and ovarian cancer syndrome, pathogenic germline variants, stomach cancer

## Abstract

**Simple Summary:**

Germline *BRCA1* and *BRCA2* pathogenic variant carriers are recognized to be at increased risk for multiple cancers including breast, ovarian, pancreatic, and prostate cancer, with risk management recommendations for these cancers included in *BRCA1/2* guidelines. Currently, it is remains uncertain whether *BRCA1/2* carriers are also at an increased risk for gastric cancer. Herein, we review the accumulating evidence that suggests *BRCA1/2* carriers are at increased risk for gastric cancer, particularly among *BRCA2* carriers. We also review existing literature addressing *BRCA1/2*-associated gastric carcinogenesis and potential avenues for therapeutic intervention. Lastly, we present gastric cancer risk management considerations for *BRCA1/2* carriers as currently no such recommendations exist.

**Abstract:**

Carriers of a pathogenic germline variant (PV) in *BRCA1* or *BRCA2* are at increased risk for a number of malignancies, including breast, ovarian, pancreatic, and prostate cancer. In this review, we discuss emerging evidence that *BRCA2* PV carriers, and likely also *BRCA1* PV carriers, are also at increased risk for gastric cancer (GC), highlighting that GC may be part of the *BRCA1/2* cancer risk spectrum. While the pathogenesis of GC among *BRCA1/2* PV carriers remains unclear, increasing evidence reveals that GCs are often enriched with mutations in homologous recombination-associated genes such as *BRCA1/2*, and that GC prognosis and response to certain therapies can depend on BRCA1/2 expression. Given the strength of data published to date, a risk management strategy for GC among *BRCA1/2* PV carriers is needed, and herein we also propose a potential strategy for GC risk management in this population. Moving forward, further study is clearly warranted to define the mechanistic relationship between *BRCA1/2* PVs and development of GC as well as to determine how GC risk management should be factored into the clinical care of *BRCA1/2* carriers.

## 1. Introduction

DNA damage resulting in double-strand breaks (DSBs) creates genomic instability and initiates the DNA damage response to allow for repair of these DSBs [1,2]. The two primary processes of DNA DSB repair are non-homologous end joining (NHEJ) and homologous recombination (HR). In NHEJ the broken ends of DNA are directly ligated [3,4], which can result in small nucleotide deletions at the DSB site or larger deletions and chromosomal rearrangement if multiple DSBs are simultaneously present [2,3,4]. Conversely, HR is less error prone as it involves the use of a homologous DNA sequence as a template to restore lost genetic information at the site of a DSB [1,2].

**Figure 1:**
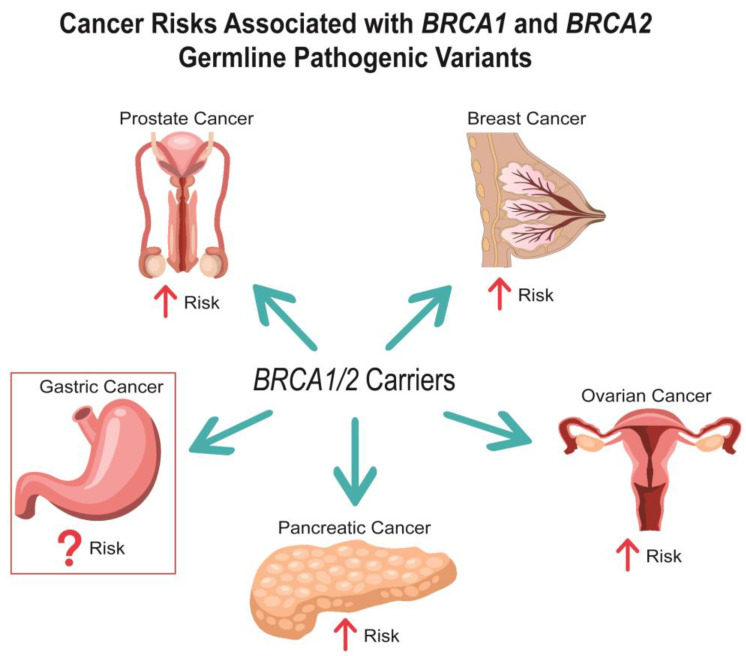
Cancer Risks Associated with *BRCA1* and *BRCA2* Germline Pathogenic Variants.

Breast cancer susceptibility gene one (*BRCA1*) and two (*BRCA2*), are well established tumor suppressor genes that play a pivotal role in promoting HR in response to DNA damage [5,6,7]. Indeed, pathogenic germline variants (PVs) in either of these genes leads to an increase in NHEJ, which can in turn promote genomic instability and tumorigenesis [2,3,4,8,9,10,11]. *BRCA1* was first described as a hereditary breast and ovarian cancer susceptibility gene in 1994, followed by *BRCA2* in 1995 [12,13]. Since then, the risks for breast and ovarian cancer in *BRCA1/2* PV carriers have been well characterized (Figure 1). Importantly, cancer risks differ between *BRCA1* and *BRCA2* PVs as well as by sex. For instance, female *BRCA1* PV carriers are at a higher risk for breast and ovarian cancer than *BRCA2* PV carriers [14]. Meanwhile, male *BRCA1/2* PV carriers are at a much lower absolute risk for breast cancer than females [15], although the relative breast cancer risk compared to the general population is higher in male versus female *BRCA1/2* carriers. In addition to breast and ovarian cancer, an increased risk for prostate cancer in *BRCA2* PV carriers as well as pancreatic cancer in both male and female *BRCA1/2* PV carriers has been recognized [15,16]. Currently, there are guidelines that outline risk management strategies for all the aforementioned cancers in *BRCA1/2* PV carriers [17,18,19,20].

There is mounting evidence that *BRCA1/2* PV carriers also have an elevated risk of gastric cancer (GC) [15,16,21,22,23,24,25,26,27,28]. Indeed, a recent study showed a cumulative risk as high as 21.3% for *BRCA1* and 19.3% for *BRCA2* PV carriers by age 85, however this study was performed in a Japanese population where risk of GC is increased at baseline [16]. Importantly, despite this potential increased GC risk, there are currently no recommended GC surveillance guidelines for *BRCA1/2* PV carriers. Furthermore, while mechanistic implications of *BRCA1/2* in GC pathogenesis remain obscure, there is evidence that GC may be enriched for mutations in genes associated with HR and confer a tumor mutation signature associated with HR deficiency [29,30,31]. Additionally, *BRCA1/2* expression levels may be prognostic for chemotherapy response in GC [29,30,31,32,33,34,35,36,37].

Given the emerging evidence for increased GC risk in *BRCA1/2* PV carriers, it is critical, and timely, that the existing literature is evaluated to help guide development of risk management strategies and identify potential areas of future investigation. Therefore, the intent of this review is 3-fold. (1) To summarize what is currently known about the risk of GC in *BRCA1/2* PV carriers. (2) Describe the evidence of HR deficiency and altered *BRCA1/2* expression in GC pathogenesis. (3) Identify potential strategies for gastric surveillance in *BRCA1/2* PV carriers.

## 2. Gastric Cancer Risk in *BRCA1/2* Carriers

Over the last three decades there have been multiple studies that examined risk of GC among *BRCA1/2* PV carriers. While the majority of these studies analyzed *BRCA1* and *BRCA2* PV carriers as separate populations, other studies evaluated *BRCA1/2* carriers as a combined cohort. When presenting the data from studies examining GC risk in *BRCA1/2* PV carriers, we have divided this data between *BRCA1* PV carriers, *BRCA2* PV carriers, and study populations comprised of both *BRCA1* and *BRCA2* PV carriers. Of note, in the majority of these *BRCA1/2* studies, individuals did not necessarily undergo multigene panel testing, and thus it is possible that some of the observed cases of GC were driven by PVs in genes increasing risk of GC other than *BRCA1/2*.

### 2.1. Gastric Cancer Risk among BRCA1 PV Carriers

Several studies have provided compelling evidence that germline *BRCA1* PVs may increase risk of GC (Table 1). In a 1999 study by Johannsson et al. [27] examining the incidence of malignant tumors in 29 Swedish families (n = 1145 relatives) with a proband carrying a germline *BRCA1* PV, incidence of GC was increased among all individuals (standardized morbidity rate (SMR) 2.76, 95% CI 1.01–6.00) and women in isolation (SMR 5.16, 95% CI 1.14–13.22), but not among men (SMR 1.43, 95% CI 0.17–5.15). In a 2001 study by Risch et al. [38] involving 649 patients in Canada with ovarian cancer, including 39 *BRCA1* PV carriers, risk of GC was increased 6-fold in first-degree relatives of *BRCA1* PV carriers compared to relatives of non-carriers (incidence 4.9% vs. 0.8%; RR 6.2, 95% CI 2.0–19). One year later, Brose et al. [39] similarly found that age-adjusted GC risk was seven times higher among *BRCA1* PV carriers (n = 483) from two U.S. centers compared to the general population (5.5% vs. 0.8%, 95% CI 3.4–7.5%). In a 2012 study by Moran et al. [23] involving 268 *BRCA1*-associated families in England, risk of GC was increased in *BRCA1*-positive families compared to the general population (RR 2.4, 95% CI 1.2–4.3), although notably this risk was driven by GC diagnosed among 1184 potential *BRCA1* PV carriers that had not undergone *BRCA1/2* testing. More recently in 2022, Li et al. [15] examined cancer risk among 3184 families in the multinational Consortium of Investigators of Modifiers of *BRCA1/2* (CIMBA) with at least one family member having a *BRCA1* PV, noting increased risk of GC among 8884 *BRCA1* PV carriers (RR 2.17, 95% CI 1.25–3.77), particularly including those less than 65 years old (RR 3.50, 95% CI 2.01–6.10). By age 80, the absolute risk of developing GC among *BRCA1* PV carriers was 0.7% (95% CI 0.3–1.7) for females and 1.6% (95% CI 0.7–4.0) for males [15]. Furthermore, in a 2022 case–control study in Japan by Momozawa et al. [16] involving 63,828 patients with at least one of 14 different cancer types and 37,086 controls, *BRCA1* PVs were again associated with increased risk of GC (OR 5.2, 95% CI 2.6–10.5). The average age of GC diagnosis was 62.3 ± 12.0 among *BRCA1* PV carriers compared to 65.7 ± 10.5 among non-carriers of *BRCA1/2* PVs (*p* = 0.14), and cumulative GC risk was 21.3% (95% CI 6.9–33.4%) by age 85 in *BRCA1* PV carriers compared to just below 5% for individuals without a *BRCA1/2* PV [16]. Notably, the high cumulative GC risk for all subsets of patients in this study likely reflects the elevated incidence of GC in East Asian countries [40]. Furthermore, sex-specific GC risks were not calculated in this study.

In contrast, there are other studies suggesting that GC risk may not be increased among germline *BRCA1* PV carriers. In a 1994 study by Ford et al. [41] of 33 families with linkage to *BRCA1* (including 464 *BRCA1* PV carriers) in North America and Western Europe in the Breast Cancer Linkage Consortium (BCLC), only one carrier was found to have GC, which did not significantly vary from what was expected (0.76 cases expected) based on population metrics (RR 1.11, *p* > 0.05). Several years later, a 2002 study comprised of 699 families in Western Europe and North America with at least one member carrying a *BRCA1* PV noted that risk of GC was not statistically significantly higher among 2245 *BRCA1* PV carriers compared to the general population (RR 1.56, 95% CI 0.91–2.68) [42]. Likewise, a 2010 study by Schlebusch et al. [26] involving South African families with history of breast and/or ovarian cancer noted that risk of GC was not increased among 26 families (n = 793 relatives) with a *BRCA1* PV compared to the general population (7 cases observed vs. 6.62 expected, *p* = 0.8829). In 2014, a study by Mersch et al. [43] similarly noted that GC risk was not increased among 613 *BRCA1* PV carriers (standardized incidence ratio (SIR) 1.736, 95% CI 0.023–9.661). Two years later, a 2016 study in Poland demonstrated that founder *BRCA1* PVs were not detected more frequently among 317 patients with GC compared to 4570 controls (0.63% vs. 0.48%; OR 1.3, 95% CI 0.3–5.6) [44]. Most recently, in a 2021 meta-analysis by Lee et al. [45], *BRCA1* PVs were not associated with increased risk of GC (RR 1.70, 95% CI 0.93–3.09) based on the collective analysis of data from five studies, all of which are cited above [23,26,38,42,43]; notably, the 2022 studies by Li et al. [15] and Momozawa et al. [16], which both noted an association between *BRCA1* PVs and increased GC risk, were published after this 2021 meta-analysis. Thus, accounting for all evidence published to date, it seems most likely that *BRCA1* PV carriers are at a modestly increased risk for GC, although further study is warranted to more clearly define this relationship. 

### 2.2. Gastric Cancer Risk among BRCA2 PV Carriers

A majority of studies have found that GC risk is increased among germline *BRCA2* PV carriers (Table 2). In a 1999 study by the BCLC involving 173 families with *BRCA2* PVs in Western Europe, the United States, and Canada (n = 3728 relatives), *BRCA2* PV carriers were at increased risk for GC compared to the general population (RR 2.59, 95% CI 1.46–4.61) [28]. Shortly thereafter, in a 2001 study involving 70 Ashkenazi Jewish patients with gastrointestinal (GI) malignancies, the *BRCA2* Ashkenazi Jewish founder PV (6174delT) was enriched among patients with GC compared to the general Ashkenazi Jewish population (5.7% vs. 1.2%; OR 5.2, 95% CI 1.2–22) [25]. The following year, Tulinius et al. [46] examined the effect of a single Icelandic founder *BRCA2* PV (999del5) on cancer risk among families of 995 breast cancer patients in Iceland, 90 of whom tested positive for the *BRCA2* founder. GC risk was increased among male first-degree relatives of *BRCA2*-positive probands (RR 2.40, 95% CI 1.29–4.05), male second-degree relatives of *BRCA2*-positive probands (RR 1.91, 95% CI 1.33–2.63), female second-degree relatives of *BRCA2*-positive probands (RR 3.08, 95% CI 2.09–4.34), and female second-degree relatives of all probands (RR 1.39, 95% CI 1.17–1.61) [46]. That same year, Jakubowska et al. [24] published a study based in Poland revealing that *BRCA2* PVs were observed more frequently among 29 families with at least one female breast cancer diagnosed before the age of 50 and one male GC diagnosed before the age of 55 compared to 248 breast-ovarian cancer families (20.7% vs. 6.9%, *p* < 0.025). In 2003, the same authorship group published a similar study revealing that *BRCA2* PVs were found more frequently in 34 Polish women with ovarian cancer and a family history of GC (mean age of GC diagnosis of 59 years, range: 33–76) compared to 75 Polish women with ovarian cancer and a family history of ovarian but not GC (23.5% vs. 4.0%; OR 7.4, 95% CI 1.8–30) [47]. Several years later, the aforementioned 2010 study by Schlebusch et al. [26] examining cancer prevalence among South African breast-ovarian cancer families revealed that prevalence of GC was increased among 43 families (n = 1264 relatives) with a *BRCA2* PV compared to the general population (24 cases observed vs. 11.17 expected, *p* = 0.0001). Along the same lines, risk of GC was increased among *BRCA2* families (RR 2.7, 95% CI 1.3–4.8) in the 2012 study by Moran et al. [23], which included 222 *BRCA2* families comprised of 517 individuals that tested positive for a *BRCA2* PV and 1009 non-tested first-degree relatives of *BRCA2* PV carriers. More recently, in the 2021 meta-analysis by Lee et al. [45], *BRCA2* PVs were associated with increased risk of GC (RR 2.15, 95% CI 1.98–2.33) based on analysis of six studies, each of which is cited in this subsection [23,26,38,43,48]. In the 2022 multinational study by Li et al. [15], which included 2157 *BRCA2* families, *BRCA2* PV carriers (n = 6095) were again found to be at increased risk of GC (RR 3.69, 95% CI 2.40–5.67). While the relative risk of GC was higher among females compared to males (6.89 vs. 2.76, *p* = 0.04), both male and female *BRCA2* PV carriers had an absolute risk of developing GC by age 80 of 3.5% [15]. Finally, *BRCA2* PVs were associated with increased risk of GC (OR 4.7, 95% CI 3.1–7.1) in the 2022 study by Momozawa et al. [16] in a Japanese population. Among *BRCA2* PV carriers, the average age of GC diagnosis was 64.5 ± 9.7, and the cumulative risk of GC was 19.3% (95% CI 11.9–26.0%) by age 85. [16]

There are only a few studies that have not found an elevated risk of GC among *BRCA2* PV carriers. In the 1999 study by Johannsson et al. [27], GC risk was not increased among 20 *BRCA2*-associated families (n = 728 relatives) compared to the general population (SMR 1.63, 95% CI 0.34–4.75). Similarly, in the 1999 study by Risch et al. [38], in which *BRCA2* PVs were identified in 21 of 649 women with ovarian cancer, no association was found between risk of GC and presence of a *BRCA2* PV (RR 2.3, 95% CI 0.30–18). Similarly, GC risk was not increased among individuals with a 50% probability of having a *BRCA2* PV (n = 1811) in a 2005 study based in the Netherlands (RR 1.2, 95% CI 0.6–2.0) [48]. Finally, in the aforementioned 2014 study by Mersch et al. [43], risk of GC was not increased among 459 *BRCA2* PV carriers compared to the general population (SIR 1.755, 95% CI 0.023–9.763). Despite the handful of studies to the contrary, the constellation of evidence cited above, highlighted by the 2021 meta-analysis by Lee et al. [45] and 2022 studies by Li et al. [15] and Momozawa et al. [16], strongly suggests that *BRCA2* PV carriers are at increased risk for GC.

### 2.3. Gastric Cancer Risk among Cohorts Comprised of BRCA1 and BRCA2 PV Carriers

Four studies analyzed risk of GC among cohorts comprised of both *BRCA1* and *BRCA2* PV carriers, with the majority noting increased GC risk in this combined population (Table 3). In a 2004 study by Bermejo et al. [49] among families with at least three generations in the Swedish Family-Cancer Database that met eligibility criteria for *BRCA1* or *BRCA2* PV testing (n = 130,487), development of GC by age 70 occurred twice as frequently in families with breast and ovarian cancer (1.88%, 95% CI 1.05–3.12%) compared to the general population (0.92%). Similarly, in a 2019 study in Korea among first- and second-degree relatives of high risk breast cancer patients (n = 2555, including 377 *BRCA1/2* PV carriers), the proportional incidence of a family history of GC was higher among *BRCA1/2* PV carriers compared to patients without a *BRCA1/2* PV (13.8% vs. 7.4%; OR 1.666, 95% CI 1.183–2.345) [50]. Most recently, in a 2020 study using data from The Cancer Genome Atlas (TCGA) and a Chinese academic center, the proportional incidence of GC among male *BRCA1/2* PV carriers with tumors (n = 294) was higher than that among non-*BRCA1/2* carrying males with tumors (n = 4577) (11.9% vs. 5.5%, *p* < 0.001) in a subgroup analysis of the TCGA population [21]. On the other hand, in a 2012 study of 238 high-risk breast cancer patients in Korea (including 49 *BRCA1/2* PV carriers), the proportional incidence of a family history of GC did not vary between patients without *BRCA1/2* PVs and *BRCA1/2* PV carriers (24.7% vs. 20.5%; RR 0.947, 95% CI 0.822–1.091) [51]. Altogether, three of the four studies noted an increased risk of GC in this combined population of *BRCA1/2* carriers, further substantiating germline *BRCA1/2* PVs as a risk factor for GC.

## 3. *BRCA1/2*-Associated Gastric Cancer

### 3.1. Classical Pathways of Gastric Carcinogenesis

The two major subtypes of gastric adenocarcinoma are the intestinal type and diffuse type, and these two subtypes have different mechanisms of carcinogenesis [52,53]. The Correa cascade is the classic mechanism for the development of intestinal type GC. Typically, in this pathway GC evolves from long-term gastric inflammation due to chronic or atrophic gastritis leading to intestinal metaplasia, followed by dysplasia, and eventually carcinoma [52]. *H. pylori* is a well-established pathogen that can promote progression through this pathway via promotion of chronic gastritis [54]. Another pathogen associated with gastric carcinogenesis is the Epstein–Barr virus [55], which has been associated with approximately 9% of GCs, including both intestinal and diffuse type [56]. In contrast to intestinal type GC, diffuse GC (DGC) occurs independent of the Correa cascade. DGC is poorly differentiated with a lack of intercellular adhesion and often histologically characterized by signet-ring cells [53].

Several studies have reported the presence of germline *BRCA1* and *BRCA2* mutations in cases of GC [22,29,31,57,58,59], as well as others that noted somatic *BRCA1/2* mutations [22,30]. To our knowledge, there are no reports investigating a mechanistic role of *BRCA1/2* mutations in GC pathogenesis. Thus, which of these classical pathways of GC pathogenesis might be relevant to *BRCA1/2*-associated GC remains to be determined.

### 3.2. Homologous Recombination Deficiency in Gastric Cancer

There is increasing evidence that GCs may be enriched for mutations in genes associated with HR [29,30,31]. A 2015 study by Alexandrov et al. reported that 7–12% of GCs assessed from TCGA, International Cancer Genome Consortium, and previously published articles, had a tumor mutation signature associated with HR deficiency [30]. Importantly, the authors noted a GC with a somatic *BRCA2* mutation that presented a HR deficient tumor mutation signature [30]. Furthermore, germline mutations in *BRCA1/2* HR pathway interaction partners, *PALB2* and *RAD51C,* were also shown to confer a tumor mutation signature enriched for HR deficiency in four cases of GC [29]. While this same study noted three cases of GC with a germline *BRCA1* mutation, the tumor mutation signatures were not reported [29]. Lastly, a study looking at 207 Japanese patients with GC noted a high frequency of germline mutations in HR associated genes [31]. Of the 207 patients, 10.6% harbored a *BRCA2* mutation, 9.7% with an *ATM* mutation, 4.3% with a *PALB2* mutation, 4.3% with a *RAD50* mutation, as well as 3.9% with a *BRCA1* mutation [31].

### 3.3. Prognostic Value of BRCA1/2 Expression in Gastric Cancer

Three different groups independently published similar findings in 2013 looking at *BRCA1* protein expression via immunohistochemistry as a potential prognostic indicator in GC [32,33,34]. Of note, these studies did not check for the presence of germline or somatic *BRCA1* mutations. Chen et al., evaluated *BRCA1* protein expression in surgically resected GC tissue from 637 patients [32]. The investigation found that lack of *BRCA1* expression was associated with poor tumor differentiation, advanced-stage disease, and decreased overall survival (OS) compared to patients with *BRCA1* expression in their GC [32]. Zhang and colleagues described similar findings, reporting that of 125 GC tissue samples 21.4% showed a loss of *BRCA1* expression [33]. Importantly, the loss of *BRCA1* expression was found to be significantly associated with DGC, a higher tumor grade, advanced clinical stage, and a lower 2-year survival rate compared to patients with positive *BRCA1* expression [33]. The findings of the previous two groups were further corroborated by Kim et al., who demonstrated that the tumors of patients with sporadic GC and low to negative *BRCA1* nuclear expression were associated with advanced-stage disease and perineural invasion [34]. Furthermore, disease-free survival (DFS) of patients with low to negative *BRCA1* expression was significantly decreased compared to patients with high expression [34]. Converse to these findings, a study by Wang et al. reported that GC tumors with high nuclear expression of *BRCA1* were associated with a worse OS [35]. However, high cytoplasmic expression was associated with a better OS [35]. These findings suggests that *BRCA1* expression and protein localization may be an informative marker of GC prognosis. Nonetheless, future studies to validate such a hypothesis are needed.

Only a few studies have evaluated *BRCA2* protein expression as a prognostic marker in GC. Wang et. al., detected *BRCA2* expression exclusively in the cytoplasm of both normal and GC tumor cells [35]. Importantly, GC tumors with high expression of cytoplasmic *BRCA2* were associated with a better OS [35]. A more recent study in 2022 was able to detect both cytoplasmic and nuclear *BRCA2* protein expression in GC tumor tissues [36]. The investigators showed that positive nuclear *BRCA2* expression was correlated with DGC and a lower OS [36]. Correlations for cytoplasmic expression of *BRCA2* were not reported [36]. Given the limited number of studies, additional investigations are needed to determine if *BRCA2* protein expression and its cellular localization has prognostic value in GC.

### 3.4. Potential Therapeutic Interventions in BRCA1/2 Associated Gastric Cancer

The emerging evidence of HR deficiency and altered expression of *BRCA1/2* proteins in GC suggests a potential role for the employment of DNA damaging chemotherapeutic agents in *BRCA1/2*-associated GC. Such approaches have been shown to be efficacious in treatment of breast and ovarian cancers of *BRCA1/2* carriers [60,61,62,63,64,65,66], as well pancreatic cancers with an inactivation of *BRCA1, BRCA2,* or *PALB2* and an HR deficiency tumor mutation signature [67].

Regarding BRCA1 expression, Chen et al. found that patients with GC and negative *BRCA1* expression saw a greater OS benefit compared to patients with positive *BRCA1* expression when administered platinum-based adjuvant chemotherapy [32]. Kim et al., demonstrated that patients with stage III GC and negative *BRCA1* nuclear expression saw a significant increase in OS and DFS when given a fluoropyrimidine combined with platinum-based adjuvant chemotherapy compared to patients with positive *BRCA1* nuclear expression [34]. Interestingly, the combined fluoropyrimidine and platinum-based adjuvant treatment seemed to confer a better OS and DFS than fluoropyrimidine alone in the stage III patients with negative *BRCA1* nuclear expression [34]. This perhaps further implicates a role for platinum-based DNA damaging agents in GC when *BRCA1* expression is reduced or absent. Lastly, Moiseyenko et al. showed that GC patients with tumor tissue samples that had low *BRCA1* transcript levels had a significantly higher overall response and clinical benefit compared to those with high *BRCA1* transcript levels when administered a combination of platinum-based agents, fluoropyrimidines, and anthracyclines [37]. However, this study did not find a significant improvement in PFS or OS in this cohort [37]. Separate from the transcript analyses, the investigators described two patients with a *BRCA1* germline PV and loss of heterozygosity (LOH) in GC tumor samples [37]. The authors note that these two patients both appeared to show an enhanced sensitivity to platinum-based chemotherapy by means of tumor size reduction [37]. Taken together, this evidence begins to implicate a potential chemotherapeutic role for DNA damaging agents such as platinum-based compounds when *BRCA1* expression is altered in GC. Of note, we were unable to identify similar studies of *BRCA2* expression and response to chemotherapy in the context of GC.

While the studies presented here suggest improved responsiveness of *BRCA1/2*-associated GCs to platinum-based intervention, other DNA damaging agents such as PARP inhibitors (PARPi) may also prove useful as PARPi have shown selective killing of *BRCA1/2* mutant tumor cells [68,69]. Additionally, clinical trials have shown promise in treatment of *BRCA1/2* PV carriers in other cancers [61,62,63,64]. Given the emerging risk of GC in germline *BRCA1/2* PV carriers, future studies and clinical trials are crucial to more clearly determine the chemotherapeutic agents that are the most efficacious in the treatment of GC when *BRCA1*/2 expression is altered and/or in the presence of germline *BRCA1/2* PVs. Furthermore, such studies involving germline *BRCA1/2* PV carriers should also take into consideration locus-specific LOH status as a study by Maxwell et al. indicated that the absence of locus-specific LOH may predict primary resistance to DNA damaging agents [70]

## 4. Gastric Surveillance Considerations in *BRCA1/2* Carriers

### 4.1. Non-Gastric Surveillance in BRCA1/2 Carriers

Before pondering the potential for GC surveillance in *BRCA1/2* PV carriers, it is relevant to first review the risk management recommendations for other malignancies associated with *BRCA1/2* PVs, including breast, ovarian, prostate, and pancreatic cancer [71,72]. For breast cancer surveillance, National Comprehensive Cancer Network (NCCN) guidelines recommend that female *BRCA1/2* PV carriers are taught breast awareness at age 18 and undergo clinical breast exams every 6–12 months starting at age 25, annual breast magnetic resonance imaging (MRI) from age 25–30, and annual mammogram and breast MRI from age 30–75, supplemented with discussion of the option of risk-reducing mastectomy [71]. Male *BRCA1/2* PV carriers are recommended to undergo annual breast exams starting at age 35, with consideration of annual mammogram starting at age 50 for men with gynecomastia [71]. For risk reduction of ovarian cancer in this population, NCCN guidelines recommend risk-reducing salpingo-oophorectomy (RRSO), typically between age 35–40 and after completion of childbearing, noting that it is reasonable to delay RRSO until age 40–45 among *BRCA2* PV carriers [71]. For prostate cancer surveillance, NCCN guidelines recommend prostate cancer screening starting at age 40 for *BRCA2* PV carriers, and that *BRCA1* PV carriers begin shared decision-making surrounding prostate-specific antigen screening at age 40 with consideration of surveillance at annual intervals [72]. Last, based on growing evidence that *BRCA1/2* PVs increase risk of pancreatic cancer, guidelines have recommended pancreatic cancer surveillance with endoscopic ultrasound (EUS) or MRI for those carriers with a first- or second-degree family member with pancreatic cancer [71,73]. However, recent data has explored pancreatic cancer surveillance in *BRCA1/2* carriers without a family history of pancreatic cancer [74,75], and newly released American Society for Gastrointestinal Endoscopy (ASGE) guidelines now recommend that all *BRCA1/2* PV carriers undergo pancreatic cancer surveillance starting at age 50 [76].

### 4.2. Gastric Surveillance in Other Hereditary Gastric Cancer Risk Syndromes

A number of hereditary cancer predisposition syndromes are associated with elevated risk of GC, which has led to a multitude of syndrome-specific guidelines for GC surveillance [77]. Patients with hereditary diffuse gastric cancer syndrome (HDGC) due to a PV in *CDH1* or *CTNNA1* have the highest lifetime cumulative risk of GC [57,78,79]. In HDGC, if gastrectomy is declined or delayed, guidelines recommend annual upper endoscopy with targeted and non-targeted gastric biopsies [78]. Patients with Lynch syndrome, who have a lifetime risk of GC of up to 9%, are recommended to undergo upper endoscopy starting at age 30–40, with biopsies of the antrum and body performed to rule out *H. pylori* and gastric intestinal metaplasia, followed by repeat upper endoscopy every 2–4 years thereafter [80]. Likewise, patients with familial adenomatous polyposis (FAP) are recommended to undergo upper endoscopy with ampulla visualization starting at age 20–25, with the interval of subsequent studies based on the Spigelman stage of duodenal polyposis or gastric pathology, which can range from 3 months to 5 years [80]. For patients with gastric adenocarcinoma and proximal polyposis of the stomach (GAPPS), who have an estimated risk of developing intestinal-type gastric adenocarcinoma of 12–25%, expert opinion recommends annual upper endoscopy starting at age 15 and consideration of risk-reducing total gastrectomy in their 30s [80,81,82]. In the setting of Peutz-Jeghers syndrome, which carries a 29% risk of GC by age 65, patients are recommended to undergo baseline upper endoscopy at age 8–10 followed by repeat study every 2–3 years, or again at age 18 and every 3 years thereafter if no characteristic polyps are found at baseline [83]. Similarly, patients with juvenile polyposis syndrome, who have a lifetime GC risk of up to 21–30%, are directed to undergo initial upper endoscopy between age 12–15 followed by repeat study every 1–3 years, or again at age 18 and every 1–3 years going forward if no polyps are found at baseline [80,83].

### 4.3. Gastric Cancer Risk Management in BRCA1/2 Carriers

Despite mounting evidence that GC risk is increased among *BRCA1/2* PV carriers, at this time *BRCA1/2* guidelines do not recommend any specific surveillance for GC. Discussion of the potential merits of surveillance and other strategies for GC risk management is certainly warranted by the organizations that develop and implement *BRCA1/2* surveillance guidelines (i.e., NCCN), and GC risk should be addressed in future guideline iterations. However, until that time, we favor the following strategy for GC risk management in *BRCA1/2* PV carriers:

***H. pylori* testing and treatment**—It currently remains unknown whether *H. pylori* plays a role in gastric carcinogenesis among *BRCA1/2* PV carriers, and it also remains uncertain whether *BRCA1/2* PV carriers have increased prevalence of *H. pylori*. However, *H. pylori* is a well-documented GC risk factor that is treatable, and as with other hereditary GC risk syndromes, such as Lynch syndrome, we believe that it would be reasonable to test for *H. pylori* (either non-invasively or via gastric biopsy) among *BRCA1/2* PV carriers at least one-time, with treatment and confirmation of eradication if *H. pylori* is present.

**Perform careful upper endoscopy concurrently when endoscopic ultrasound is performed for pancreatic cancer surveillance**—As current ASGE guidelines recommend that all *BRCA1/2* PV carriers initiate pancreatic cancer surveillance at age 50, it is likely that an increasing number of *BRCA1/2* PV carriers will be undergoing EUS for pancreatic cancer surveillance [76]. We believe that if a *BRCA1/2* PV carrier is already undergoing an EUS for pancreatic cancer surveillance, a standard upper endoscopy should also be performed concurrently with careful inspection for upper GI neoplasia.

**Low threshold for upper endoscopy for upper GI symptom investigation**—In other hereditary syndromes associated with increased GC risk, the threshold of most clinicians for performing upper endoscopy for evaluation of upper GI symptoms is lower than for the general population. We would recommend that the threshold for upper endoscopy in *BRCA1/2* PV carriers with upper GI symptoms be similarly low.

**Consider regular gastric surveillance in *BRCA1/2* PV carriers with a family history of GC**—If a *BRCA1/2* PV carrier has a family history of GC in a carrier of the *BRCA1/2* PV, we believe that a baseline surveillance upper endoscopy can be offered at age 50, or 10 years prior to the youngest GC diagnosis, and then can be repeated at an interval of 3 years.

While the above considerations are based on our professional opinion, further study and expert discussion is urgently warranted to construct an optimal, evidence-based risk management strategy for GC risk among *BRCA1/2* PV carriers, particularly as the incidence of GC in this population will likely increase over time as improvements in the detection and treatment of other at-risk malignancies (i.e., breast, ovarian, prostate, pancreatic) prolongs the overall lifespan of this cohort.

## 5. Conclusions

A growing body of evidence indicates that risk of GC is increased among *BRCA2* PV carriers, and likely also among *BRCA1* PV carriers. Furthermore, increasing evidence implicates a potential role of HR associated genes (e.g., *BRCA1/2*) and their altered expression in GC pathogenesis, which may have important prognostic and therapeutic implications. Moreover, the strength of the data published to date warrants that the organizations charged with developing cancer surveillance guidelines consider providing an evidence-based risk management strategy for GC among *BRCA1/2* PV carriers. Altogether, the emerging clinical evidence and remaining questions surrounding pathogenesis and optimal risk management strategies make GC among *BRCA1/2* PV carriers an intriguing area for further research.

## Figures and Tables

**Table 1:** Select Studies Reporting Gastric Cancer Risk in *BRCA1* Carriers.

Author	Year	Population Location	Patient Population	Comparator Cohort	Risk Estimates	Gastric Cancer Risk Increased?
**Gastric Cancer Risk in *BRCA1* PV Carriers**						
Ford et al. [41]	1994	North America and Western Europe	464 *BRCA1* PV carriers	General Population	1 observed case vs. 0.76 cases expected; RR 1.11, *p* > 0.05	No
Johannsson et al. [27]	1999	Sweden	1145 relatives from 29 families with a proband with a *BRCA1* PV	General Population	All: SMR 2.76, 95% CI 1.01–6.00; F: SMR 5.16, 95% CI 1.14–13.22; M: SMR 1.43, 95% CI 0.17–5.15	Yes
Risch et al. [38]	2001	Canada	39 *BRCA1* PV carriers and 291 FDRs	4378 FDRs of ovarian cancer patients without *BRCA1* or *BRCA2* PVs	Incidence: 4.9% vs. 0.8%; RR 6.2, 95% CI 2.0–19	Yes
Brose et al. [39]	2002	United States	483 *BRCA1* PV carriers	General Population	Age-adjusted lifetime risk: 5.5% vs. 0.8%, 95% CI 3.4–7.5%	Yes
Thompson et al. [42]	2002	North America and Western Europe	2245 *BRCA1* PV carriers	General Population	RR 1.56, 95% CI 0.91–2.68	No
Schlebusch et al. [26]	2010	South Africa	793 individuals from 26 families with a *BRCA1* PV	General Population	7 cases observed vs. 6.62 cases expected, *p* = 0.8829	No
Moran et al. [23]	2012	England	631 *BRCA1* PV carriers and 1184 FDRs from 268 families with a *BRCA1* PV	General Population	RR 2.4, 95% CI 1.2–4.3	Yes
Mersch et al. [43]	2014	United States	613 *BRCA1* PV carriers	General Population	SIR 1.736, 95% CI 0.023–9.661	No
Li et al. [15]	2022	Multinational (>10 countries)	8884 *BRCA1* PV carriers	General Population	RR 2.17, 95% CI 1.25–3.77	Yes
***BRCA1* PVs amongst gastric cancer patients**						
Ławniczak et al. [44]	2016	Poland	317 patients with GC	4570 controls	Mutation rate: 0.63% vs. 0.48%; OR 1.3, 95% CI 0.3–5.6	No
Momozawa et al. [16]	2022	Japan	10,705 cases of GC	37,086 controls	OR 5.2, 95% CI 2.6–10.5	Yes
**Meta-analysis**						
Lee et al. [45]	2021	North America, Western Europe, South Africa	Meta-analysis, including 5 studies pertaining to *BRCA1* PVs, all of which are cited in this sub-section of Table 1 [23,28,38,42,43]	Varied by study	*BRCA1* PVs were not associated with increased risk of GC (RR 1.70, 95% CI 0.93–3.09)	No

Abbreviations: CI—confidence interval; F—female; FDR—first degree relative; GC—gastric cancer; M—male; OR—odds ratio; PV—pathogenic variant; RR—relative risk; SIR—standardized incidence ratio; SMR—standardized morbidity rate.

**Table 2:** Select Studies Reporting Gastric Cancer Risk in *BRCA2* Carriers.

Author	Year	Population Location	Patient Population	Comparator Cohort	Risk Estimates	Gastric Cancer Risk Increased?
**Gastric Cancer Risk in *BRCA2* PV Carriers**						
Breast Cancer Linkage Consortium [28]	1999	Europe and North America	1152 confirmed or probable *BRCA2* PV carriers from 173 families *	General Population	RR 2.59, 95% CI 1.46–4.61	Yes
Johannsson et al. [27]	1999	Sweden	728 relatives from 20 families with a proband with a *BRCA2* PV	General Population	All: SMR 1.63, 95% CI 0.34–4.75; F: SMR 1.37, 95% CI 0.03–7.64; M: 1.79, 0.22–6.48	No
Risch et al. [38]	2001	Canada	21 *BRCA2* PV carriers and 160 FDRs	4378 FDRs of ovarian cancer patients without *BRCA1* or *BRCA2* PVs	Incidence: 1.8% vs. 0.80%; RR 2.3, 95% CI 0.30–18	No
Tulinius et al. [46]	2002	Iceland	90 families with a proband with a *BRCA2* PV	General Population	F FDRs: RR 1.78, 95% CI 0.57–4.10; F SDRs: RR 3.08, 95% CI 2.09–4.34; M FDRs: RR 2.40, 95% CI 1.29–4.05; M SDRs: RR 1.91, 95% CI 1.33–2.63	Yes
van Asperen et al. [48]	2005	Netherlands	1811 individuals with a 50% probability of having a *BRCA2* PV	General Population	RR 1.2, 95% CI 0.6–2.0	No
Schlebusch et al. [26]	2010	South Africa	1264 individuals from 43 families with a *BRCA2* PV	General Population	24 cases observed vs. 11.17 cases expected, *p* = 0.0001	Yes
Moran et al. [23]	2012	England	517 *BRCA2* PV carriers and 1009 FDRs from 222 families with a *BRCA2* PV	General Population	RR 2.7, 95% CI 1.3–4.8	Yes
Mersch et al. [43]	2014	United States	459 *BRCA2* PV carriers	General Population	SIR 1.755, 95% CI 0.023–9.763	No
Li et al. [15]	2022	Multinational (>10 countries)	6095 *BRCA2* PV carriers	General Population	RR 3.69, 95% CI 2.40–5.67	Yes
***BRCA2* PVs amongst gastric cancer patients**						
Figer et al. [25]	2001	Israel	35 Ashkenazi Jewish patients with GC	General Ashkenazi Jewish Population	Mutation rate: 5.7% vs. 1.2%; OR 5.2, 95% CI 1.2–22	Yes
Jakubowska et al. [24]	2002	Poland	29 breast cancer patients from families with ≥1 female breast cancer diagnosed before age 50 and ≥1 male GC diagnosed before age 55	248 breast-ovarian cancer families	Mutation rate: 20.7% vs. 6.9%, *p* < 0.025	Yes
Jakubowska et al. [47]	2003	Poland	34 women with ovarian cancer and family history of GC	75 women with ovarian cancer and family history of ovarian cancer but not GC	Mutation rate: 23.5% vs. 4.0%; OR 7.4, 95% CI 1.8–30	Yes
Momozawa et al. [16]	2022	Japan	10,705 cases of GC	37,086 controls	OR 4.7, 95% CI 3.1–7.1	Yes
**Meta-analysis**						
Lee et al. [45]	2021	North America, Western Europe, South Africa	Meta-analysis, including 6 studies pertaining to *BRCA2* PVs, all of which are cited in this sub-section of Table 2 [23,25,28,38,43,49]	Varied by study	*BRCA2* PVs were associated with increased risk of GC (RR 2.15, 95% CI 1.98–2.33)	Yes

* In a cohort of families with *BRCA2* PVs, probable *BRCA2* PV carriers were defined as men with breast cancer, females with breast cancer diagnosed < 60 years old, and females with ovarian cancer (excluding known non-carriers). Abbreviations: CI—confidence interval; F—female; FDR—first degree relative; GC—gastric cancer; M—male; OR—odds ratio; PV—pathogenic variant; RR—relative risk; SDR—second degree relative; SIR—standardized incidence ratio; SMR—standardized morbidity rate.

**Table 3:** Select Studies Reporting Gastric Cancer Risk in Combined Populations of *BRCA1* and *BRCA2* Carriers.

Author	Year	Population Location	Patient Population	Comparator Cohort	Risk Estimates	Gastric Cancer Risk Increased?
**Gastric Cancer Risk in *BRCA1/2* PV Carriers**						
Bermejo et al. [49]	2004	Sweden	10,359 individuals from families with breast and ovarian cancer	General Population	Incidence: 1.88% (95% CI 1.05–3.12%) vs. 0.92%	Yes
Noh et al. [51]	2012	Korea	49 *BRCA1/2* PV carriers	189 high-risk breast cancer patients without *BRCA1/2* PVs	Proportional incidence of a family history of GC: 24.7% for comparator cohort vs. 20.5% for *BRCA1/2* PV carriers; RR 0.947, 95% CI 0.822–1.091	No
Kim et al. [50]	2019	Korea	377 *BRCA1/2* PV carriers	2178 high-risk breast cancer patients without *BRCA1/2* PVs	Proportional incidence of a family history of GC: 13.8% vs. 7.4%; OR 1.666, 95% CI 1.183–2.345	Yes
Sun et al. [21]	2020	Multinational (>10 countries)	294 male *BRCA1/2* PV carriers with tumors	4577 male patients with tumors who were not carriers of *BRCA1/2* PVs	Proportional incidence of GC: 11.9% vs. 5.5%, *p* < 0.001	Yes

Abbreviations: CI—confidence interval; GC—gastric cancer; OR—odds ratio; PV—pathogenic variant; RR—relative risk.
